# Shugoshin1 May Play Important Roles in Separation of Homologous Chromosomes and Sister Chromatids during Mouse Oocyte Meiosis

**DOI:** 10.1371/journal.pone.0003516

**Published:** 2008-10-24

**Authors:** Shen Yin, Jun-Shu Ai, Li-Hong Shi, Liang Wei, Ju Yuan, Ying-Chun Ouyang, Yi Hou, Da-Yuan Chen, Heide Schatten, Qing-Yuan Sun

**Affiliations:** 1 State Key Laboratory of Reproductive Biology, Institute of Zoology, Chinese Academy of Sciences, Beijing, China; 2 Graduate School, Chinese Academy of Sciences, Beijing, China; 3 Department of Veterinary Pathobiology, University of Missouri-Columbia, Columbia, Missouri, United States of America; Istituto Dermopatico dell'Immacolata, Italy

## Abstract

**Background:**

Homologous chromosomes separate in meiosis I and sister chromatids separate in meiosis II, generating haploid gametes. To address the question why sister chromatids do not separate in meiosis I, we explored the roles of Shogoshin1 (Sgo1) in chromosome separation during oocyte meiosis.

**Methodology/Principal Findings:**

Sgo1 function was evaluated by exogenous overexpression to enhance its roles and RNAi to suppress its roles during two meioses of mouse oocytes. Immunocytochemistry and chromosome spread were used to evaluate phenotypes. The exogenous Sgo1 overexpression kept homologous chromosomes and sister chromatids not to separate in meiosis I and meiosis II, respectively, while the Sgo1 RNAi promoted premature separation of sister chromatids.

**Conclusions:**

Our results reveal that prevention of premature separation of sister chromatids in meiosis I requires the retention of centromeric Sgo1, while normal separation of sister chromatids in meiosis II requires loss of centromeric Sgo1.

## Introduction

One of the major differences between meiosis and mitosis is that the former consists of two consecutive rounds of chromosome separation with only one round of DNA replication, in which chromosome number is reduced to half to produce haploid gametes. Errors in this process result in aneuploidy [1]. It is the homologous chromosomes that separate from each other during the first meiosis (meiosis I) while sister chromatids segregate during the second meiosis (meiosis II). Why do sister chromatids not separate in meiosis I? It is thought that one linkage between sister chromatids exists in meiosis I while it is not present in meiosis II. Previous reports have demonstrated that the multi-subunit complex, cohesin, is responsible for the linkage [2]. Rec8, a counterpart of Scc1/Rad21 in mitosis, is the most important meiotic-specific cohesion protein [3], [4]. The removal of Rec8 along chromosome arms triggers segregation of homologs during meiosis I. However, Rec8 localized around centromeres is not degraded during meiosis I, allowing sister chromatids to be moved to the same spindle pole [5]–[8]. Whatever is responsible for preventing centromeric Rec8 from degradation in meiosis I?

The Shugoshin (Sgo) family of proteins has been demonstrated to protect centromeric cohesion during mitosis and meiosis in fission yeast [9]. Many results from mitosis have confirmed that Sgo is required for protection of cohesion [10]–[13]. Moreover, Sgo collaborates with protein phosphatase 2A to protect cohesin [14]–[16]. In addition, Sgo may sense the loss of tension at the centromere to generate a spindle checkpoint signal [17], [18]. The previous work on Sgo1 was mainly focused on mitosis or meiosis of yeast [19] and maize [20], but little is known about its role in mammalian meiosis [21]. Here, we are trying to address Sgo1's roles in chromosome separation by exogenous protein overexpression to enhance, or by RNAi to suppress Sgo1 function during two meioses of mouse oocytes. The results imply that Sgo1 holds sister chromatids together in anaphase of first meiosis (AI) and that loss of Sgo1 causes chromatid separation in anaphase of second meiosis (AII) at the correct time.

## Results and Discussion

### Subcellular distribution of Sgo1 during mouse oocyte meiosis

**Figure 1:**
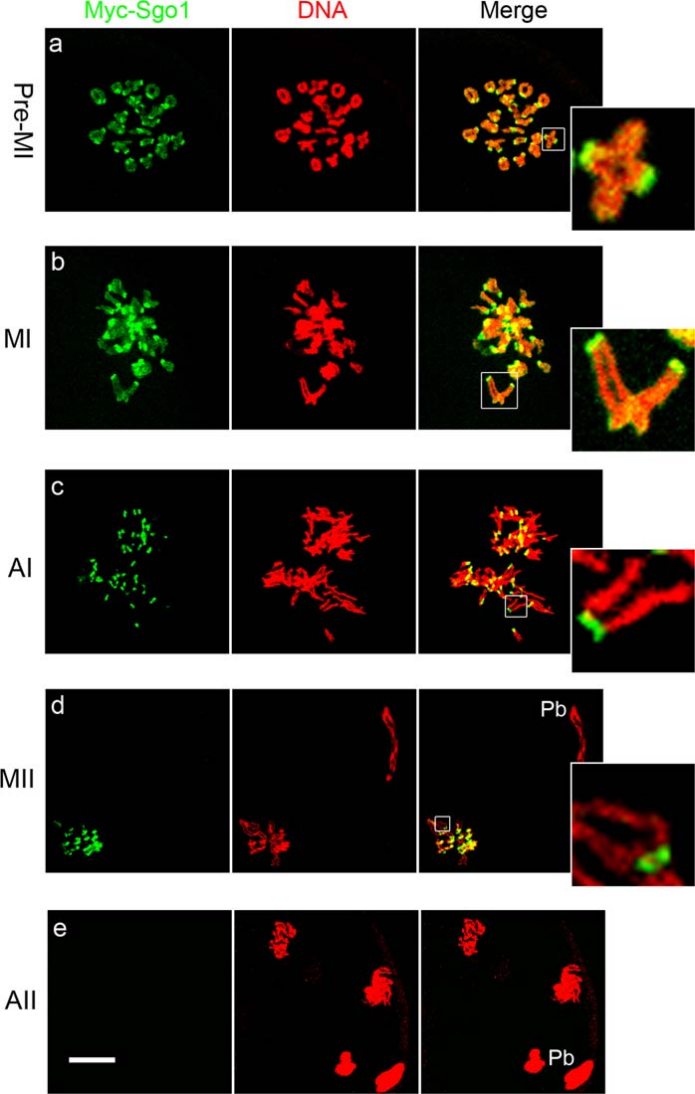
Localization of Myc-Sgo1 during mouse oocyte meiotic maturation. Prominent Myc-Sgo1 signals were observed at the centromeres of sister chromatids and faint Myc-Sgo1 signals on chromosome arms in pre-MI and MI. In AI, Myc-Sgo1 only displayed strong signals at the centromeres of sister chromatids until MII. After AII, no Myc-Sgo1 signals were observed on chromosomes. Double staining of Myc-Sgo1 (green) and DNA (red). Magnifications of the boxed regions are shown. Pb = polar body. Bar = 10 µm.

To characterize the roles of Sgo1 in chromosome separation during mouse oocyte meiosis, due to the lack of working antibody in mice, low concentration of (≤0.4 mg/ml) Myc_6_-Sgo1 mRNA was injected into oocytes to examine the dynamics of Sgo1 localization at metaphase and anaphase of both meiotic divisions. Then anti-myc-FITC antibody was used for immunofluorescence. The same amount of Myc_6_ mRNA was injected as control, but no specific signals were found. All 40 chromosomes of mouse oocytes are telocentric, so homologs form bivalents in meiosis I while sister chromatids form univalents in meiosis II [22]. As shown clearly in Fig. 1A, in pre-metaphase of meiosis I (pre-MI), synaptic homologous chromosomes turned into 20 bivalents, which have a strong Sgo1 staining in centromeres and a little faint staining along the chromosome arms (Fig. 1a). In metaphase of the first meiosis (MI), homologous chromosomes are aligned at the spindle's equator, and sister chromatids of one homolog are pulled towards the same spindle pole. Prominent Sgo1 staining was always observed at centeomeres while faint Sgo1 staining on chromosome arms (Fig. 1b). During the anaphase of the first meiosis (AI), Sgo1 signals were only detected on the centromeres of sister chromatids, until up to the metaphase of the second meiosis (MII), while no sgo1 signals were observed on arm (Fig. 1c and d, insets). After chemical activation of MII oocytes, however, Sgo1 was no longer detected on the centromeres of separated single chromosomes in the anaphase of the second meiosis (AII) (Fig. 1e). The localization of Sgo1 on centromeres of sister chromatids during mouse oocyte meiosis was completely similar to that of Rec8 in meiosis [4]–[6], [8]. It is possible that Sgo1 on centromeres holds sister chromatids together during first meiosis, and sister chromatids can not separate apart but are pulled together as one homolog towards the same spindle pole instead (Fig. 1c, inset). The loss of Sgo1 on centromeres of sister chromatids in AII stage and its concurrence with the separation of sister chromatids implies that loss of Sgo1 from centromeres is related to the separation of sister chromatids in second meiosis.

### Exogenous overexpression of Sgo1 in meiosis II held sister chromatids together

**Figure 2:**
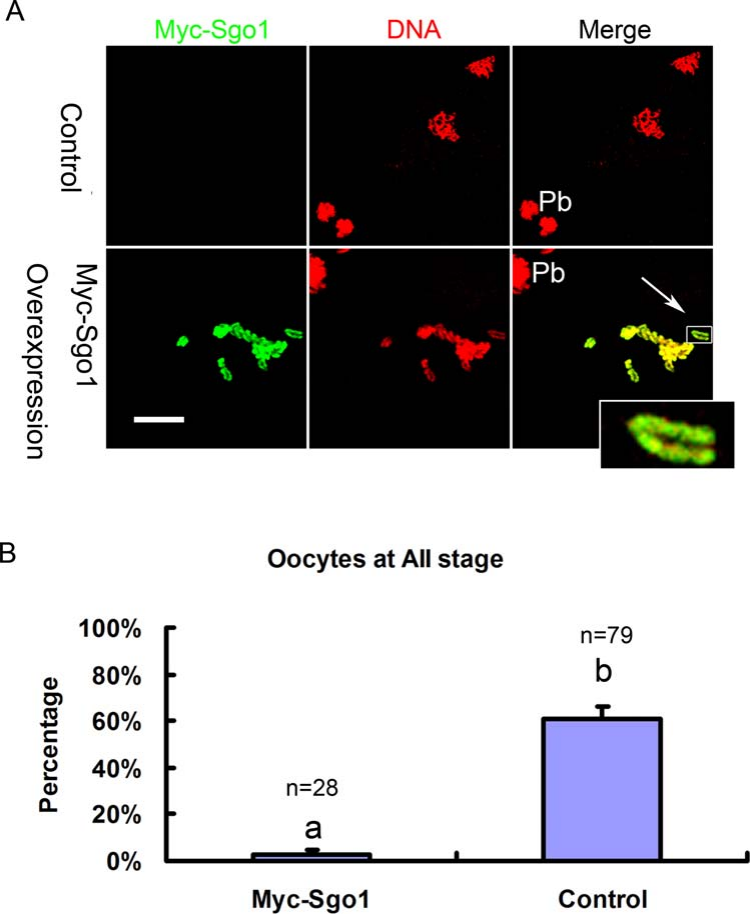
Overexpression of Myc-Sgo1 in MII holds sister chromatids together. (A) At meiosis II, sister chromatids normally separate apart in control while fail to separate apart in Myc-Sgo1overexpression. Double staining of Myc-Sgo1 (green) and DNA (red). Magnifications of the boxed regions are shown. Pb = polar body. Bar = 10 µm. (B) Effect of Myc-Sgo1 overexpression in MII oocytes. Percentage of oocytes with normal separation of sister chromatids in AII stage is shown by mean±SE. Different superscripts indicate statistical difference (P<0.01).

To confirm the hypothesis that the loss of Sgo1 is required for the separation of sister chromatids in AII, Sgo1 overexpression experiments were performed in MII oocytes. MII oocytes were injected with high concentrations (≥2.5 mg/ml) of Myc6-Sgo1 mRNA and cultured to AI stage (1 hour after chemical activation). The resulting images revealed that exogenous overexpressed Myc-Sgo1 along the arms of sister chromatids attached them tightly so that sister chromatids could not separate apart (Fig. 2A, arrow), whereas in control the sister chromatids separated apart normally. As shown in Fig. 2B, sister chromatid separation was observed in only 2.5±2.5% of oocytes in Myc signal-positive oocytes, whereas in the control the ratio was 61.1±5.2% (P<0.01). The overexpression of Myc6-Sgo1 blocked the initiation of AII and kept the oocytes at MII. As long as the overexpressed Myc-Sgo1 existed, the oocytes could not overcome the MII arrest. Can inactivation of either the spindle checkpoint or PP2A help oocytes overcome the prolonged arrest? The spindle checkpoint is not required for establishing or maintaining the CSF arrest in mouse oocytes, in contrast to frog eggs [23]. The PP2A activity is important for Emil2, which is required for maintenance of CSF arrest in frog eggs, [24], [25], while in mouse oocytes, such a function is not reported. It is implied that in exogenous Sgo1-overexpressed oocytes sister chromatids failed to separate, possibly because excessive Sgo1 protects linkage between sister chromatids from cleavage.

### Exogenous overexpression of Sgo1 in meiosis I glued homologues tightly together

**Figure 3:**
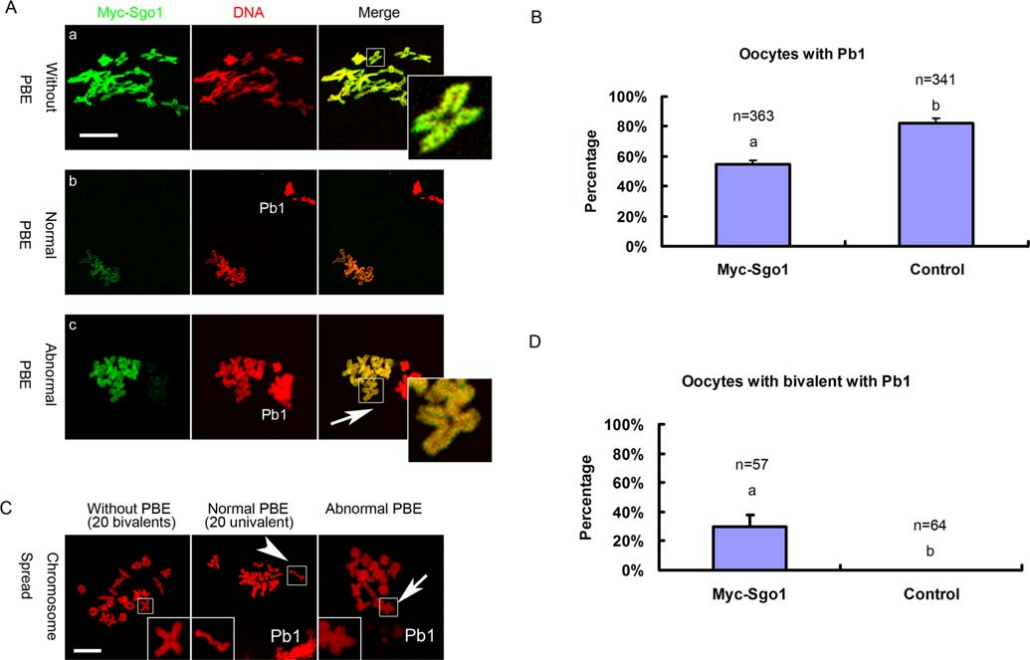
Overexpression of Sgo1 in meiosis I glues homologues tightly together. (A) At meiosis I, overexpression of Myc-Sgo1 holds homologous chromosomes together and prevents them from separating apart. The upper panel shows that homologous chromosomes can not separate and the oocytes can not extrude Pb1 at meiosis I. The lower panel shows that the oocytes can extrude Pb1 but homologous chromosomes can not separate. Double staining of Myc-Sgo1 (green) and DNA (red). (B) Effect of Myc-Sgo1 overexpression in GV oocytes. Percentage of oocytes with Pb1 is shown by mean±SE. Different superscripts indicate statistical difference (P<0.05). (C) Chromosome spreads were performed in oocytes after Myc-Sgo1 overexpression. Arrow and arrow head indicate bivalents (homologous chromosome) or univalents (sister chromatids) in oocytes with Pb1, respectively. In abnormal oocytes, bivalents are instead of univalents. (D) Percentage of Pb1-extruded oocytes containing bivalents is shown by mean±SE. Different superscripts indicate statistical difference (P<0.01). Magnifications of the boxed regions are shown. Pb1 = the first polar body. PBE = the extrusion of polar body. Bar = 10 µm.

To examine the roles of Sgo1 in meiosis I, oocytes at the germinal vesicle (GV) stage were injected with high concentrations (≥2.5 mg/ml) of Myc_6_-Sgo1 mRNA and then arrested at GV stage by 2.5 µM milrinone for 2 hours. After 14 hours of culture in milrinone-free medium, exogenous Sgo1 overexpression significantly decreased extrusion of the first polar body (PBE), from 82.2±3.3% in the control to 55±2.4% in the injection group (P<0.05) (Fig. 3B). Then we collected all oocytes and divided them into two groups for immunofluorescence microscopy, based on positive or negative PBE. In oocytes without PBE, overexpressed Sgo1 was detected on all centromeres and arms of chromosomes like an “adhesive coat”, which kept homologous chromosomes as one entity and they did not separate apart (Fig. 3Aa). A typical bivalent shape displayed non-disjunctioned homologous chromosomes, which were connected through arm regions (Fig. 3Aa, inset). Very notably, in some oocytes with PBE, which indicates that the APC/C activity and onset of anaphase are normal, bivalents consisting of homologous chromosomes were seen instead of univalents as in the control (Fig. 3Ac, arrow). To verify the observation in detail, we only collected the oocytes with PBE from both control and the Sgo1-overexpression group to perform chromosome spreads. Typical results showed that pairs of sister chromatids (20 pairs) were observed in the control oocytes (Fig. 3C, arrow head). However, in Sgo1-overexpressed oocytes with first polar body (pb1) extrusion, bivalents consisting of pairs of homologous chromosomes were observed in the oocytes (Fig. 3C, arrow). In the Sgo1 overexpression group, 30±8% of the oocytes exhibited abnormal bivalents while in the control absolutely no such oocytes were observed (P<0.01) (Fig. 3D). In this case, homologous chromosomes could not separate apart and the ultimate produced gamete would be aneuploid, which points to a significant cause for genetic disorder. Therefore, it is Sgo1 located along the arms of homologous chromosomes that attaches these chromosomes tightly together and the non-disjunctioned homologous chromosomes (bivalents) are pulled towards the same spindle poles as a whole. It is suggested that loss of Sgo1 along the arms of homologous chromosomes in meiosis I is required for homologous chromosome separation, and that Sgo1 at the centromere of sister chromatids protects them from separation until AII.

### Sgo1 prevents the separation of sister chromatids in meiosis I

**Figure 4:**
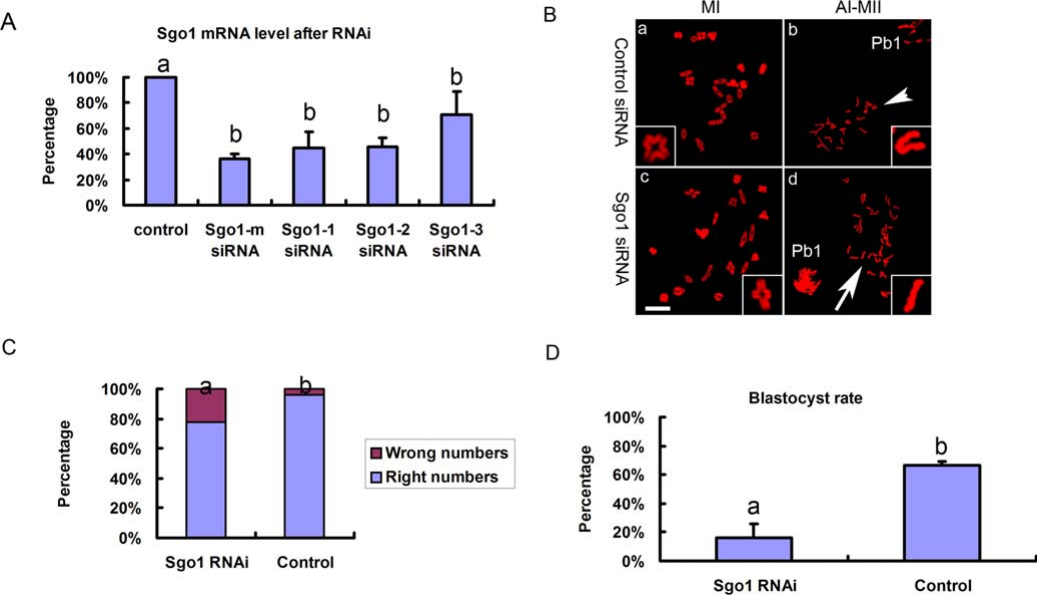
RNAi of Sgo1. (A) The efficiency of siRNAs of Sgo1. Analysis of relative gene expression was measured by using real-time quantitative PCR and the 2(-Delta Delta C(T)) method. The relative mRNA of Sgo1 compared with control (100%) is shown by mean±SE. Different superscripts indicate statistical difference (P<0.05). (B) Chromosome spreads were performed in oocytes that had been cultured for 8 h (MI) and 9h–14h (AI-MII) for maturation after siRNA treatment. In MI stage, there are 20 bivalents in control or Sgo1 RNAi. However, after MI and before initiation of AII, there are 20 univalents (arrow head) in control while 40 single chromosomes (arrow) in Sgo1 RNAi oocytes. A typical chromosome in each chromosome spread is magnified in the inset. Pb1 = the first polar body. Bar = 10 µm. (C) The percentage of oocyets with incorrect numbers of chromosomes when cultured to AI after RNAi treatment. Different superscripts indicate statistical difference (P<0.05). (D) Oocytes with Sgo1 RNAi treatment have lower ability to develop to blastocysts. Sgo1 RNAi was performed in zygotes and these zygotes were cultured for 4.5 days to observe the blastocysts. Percentage of blastocysts is shown by mean±SE. Different superscripts indicate statistical difference (P<0.01).

As exogenous expression of Sgo1 was able to hold homologous chromosomes and sister chromatids tightly together, we were curious about the effects of RNAi. We employed RNAi to delete Sgo1 to analyze separation of homologs and sister chromatids during meiosis. SiRNAs against Sgo1 was injected into oocytes at GV stage. Then the oocytes were arrested at GV by 2.5 µM milrinone for 24 hours before meiosis resumption. We confirmed the long-time GV-arrested oocytes' quality by morphological evaluation under the optical microscope and chromosome spread. The ratio of germinal vesicle breakdown (GVBD) and extrusion of the first polar body (PBE) was not statistically different from normal cultured oocytes without inhibition (data not shown). In addition, the chromosome spread images also did not show misaligned chromosomes. The efficiency of Sgo1 siRNAs was measured by real-time quantitative PCR and the 2(-Delta Delta C(T)) Method. The results indicated that the amount of Sgo1 mRNA was largely reduced (Fig. 4A). According to the results, we chose Sgo1-m siRNA to deplete Sgo1 for subsequent experiments.

The oocytes injected with Sgo1-m siRNA were cultured to MI (8h) or MII (14h) to perform chromosome spreads. The ratio (81.7±3%, n = 192) of PBE in Sgo1-RNAi oocytes was not distinctly different from the control (85.6±2.3%, n = 209). The chromosome spreads showed that depletion of Sgo1 did not affect the bivalents in MI stage (Fig. 4Bc). After MI, however, sister chromatids prematurely separated apart so that the number of chromosomes was nearly doubled, namely 20 pairs of sister chromosomes (Fig. 4Bb) turned into 40 single chromatids (Fig. 4Bd, arrow). Considering the mechanical stress during chromosome spreading, the number of chromosomes less than 25 was counted as normal. Thus, in Sgo1-RNAi oocytes, the frequency of oocytes showing disordered chromosome alignment was higher than that in control (P<0.05) (Fig. 4C). The incorrect numbers of chromosomes could be related to the premature separation of sister chromatids after MI stage, which was consistent with the results from yeast. The low frequency could be due to the inefficiency of RNAi in oocytes, because the cell cycle of oocytes is more static than that of somatic cell lines, and the RNAi effects might not be as good as in somatic cell lines.

Our RNAi results are like those most recently reported by Lee et al [21]. We had expected that some single chromatids would be found instead of bivalents in Sgo1-RNAi oocytes at MI stage, however, no abnormal chromatid separation was observed at the MI stage (Fig. 4Bc). Lee et al had proposed that Sgo2 alone plays a predominant role in protecting centromeric cohesion in meiosis I in oocytes, whereas Sgo1 is mostly, if not entirely, dispensable for this function [21]. Llano et al also supported that Sgo2 is essential for the completion of meiosis but not for mitotic cell division in mice [26]. However, here we stress the Sgo1's roles. In the MI stage, homologous chromosomes could form bivalents by chiasma, and separase-mediated cleavage of Rec8 did not occur until the onset of AI [17], [27]. No single chromatid but bivalent was found in Sgo1-RNAi oocytes even though Sgo1 was depleted at the MI stage (Fig. 4Bc). Then the oocytes proceeded AI stage. During this process, homologous chromosomes were pulled towards opposite spindle pole. Centromeres between sister chromatids lost the protection from Sgo1 and linkage between chromatids was degraded, and thus single chromatids instead of univalents was found (Fig. 4Bd, arrow). Until MII, the oocytes always showed premature separated chromatids, while in control, sister chromaitids as univalents did not separate (Fig. 4Bb). Based on our results regarding exogenous Sgo1 overexpression and RNAi, we conclude that Sgo1 plays an important role in protecting the linkage between sister chromatids during meiosis I in oocytes.

We also injected Sgo1-m siRNA into zygotes and cultured them for 4.5 days to examine the blastocyst rate. The blastocyst ratio (16±10.1%, n = 49) of the Sgo1 RNAi groups was significantly lower than that of the control groups (66.4±2.6%, n = 45) (P<0.01) (Fig. 3B). Sgo1 is therefore essential in setting up the meiotic pattern of chromosome segregation. If regarded it as true that the conservation of Sgo1 across species, mutations in human Sgo1 might induce chromosome-segregation defects in meiosis, which would lead to genetic imbalances. Many diseases are from it, such as infertility of spontaneous abortion, or to debilitating trisomies such as Down's syndrome.

In conclusion, prevention of premature separation of sister chromatids in meiosis I requires retention of centromeric Sgo1, while normal separation of sister chromatids in meiosis II requires loss of centromeric Sgo1. Given that Sgo1 is highly conserved among species, mutations in human Sgo1 might increase the frequency of aneuploidy in gametes, which would be a significant cause for genetic disorders.

## Materials and Methods

All chemicals and medium were purchased from Sigma Chemical Company (St. Louis, MO) unless stated otherwise.

### Oocyte collection and culture

ICR mice care and handling were conducted in accordance with policies promulgated by the Ethics Committee of the Institute of Zoology, Chinese Academy of Sciences. The oocytes were collected in M2 medium supplemented with 2.5 µM milrinone [28] to keep them at germinal vesicle (GV) stage. After specific treatment, oocytes were washed thoroughly and cultured in M16 supplemented with 10% fetal bovine serum (FBS) (Gibco) to (MI (8 hours) or MII (14 hours). MII-stage oocytes were released from CSF arrest by using 10 mM SrCl2 in Ca^2+^/Mg^2+^-free CZB. For *in vivo* zygote collection, one female was placed in a cage with one stud male after hCG injection, and the zygotes were collected the next morning. After microinjection, the zygotes were cultured in KSOM for 4.5 days to examine blastocyst rates.

### Sgo1 plasmid construction

Total RNA was extracted from 100 mouse GV oocytes using RNeasy micro purification kit (Qiagen), and the first strand cDNA was generated with cDNA synthesis kit (Takara), using poly (dT) primers. The following two nested primers were used to clone the full length of Sgo1 cDNA by PCR. F1: GGCCGAGATGAATTTCACTATG, R1: GGTCCACGACAGTGCTATTATTC, F2: AGCCCAAGCATAAATCTATGAC, R2: GATCCTCACCCACTTATGTCTTAC. To detect the expressed protein, the Sgo1 cDNA was then NH_2_-terminally Myc_6_-tagged. For *in vitro* transcription reactions, the Myc_6_-Sgo1 cDNA was subcloned into the modified pRN3p vector (a gift from Dr. Jie Na, Harvard University), which has a globin 3′ UTR plus a short poly A tail.

### RNA synthesis

The Myc_6_-Sgo1-pRN3p plasmid was linearized by *Sfi*I and purified by gel extraction kit (Qiagen). T3 message machine (Ambion) was used for producing capped mRNA which was purified using the RNeasy cleanup kit (Qiagen). The concentration was detected by Beckman DU 530 Analyzer, and diluted into low concentration (0.4 mg/ml) for localization tract or high concentration (2.5 mg/ml) for overexpression of protein.

### Microinjection of Myc_6_-Sgo1 mRNA or Sgo1 siRNAs

Microinjections were performed using an Eppendorf microinjector (Hamburg, Germany) and completed within 30 minutes. For Myc_6_-Sgo1 expression, 0.4 mg/ml (or 2.5 mg/ml for overexpression) Myc_6_-Sgo1 mRNA solution was injected into cytoplasm of GV stage oocytes. Oocytes were arrested at GV stage in 2.5 µM milrinone for 2 hours. The same amount of H_2_O or Myc_6_ mRNA (virtually no discrepancy was obtained from them) was injected as control. Each experiment consisted of three separate and replicate groups and approximately 100 oocytes were injected in each group. The ratio of GVBD or PBE was counted under an inverted optical microscope. For MII oocyte overexpression experiments, oocyte-cumulus complexes (COC) were cultured in M16 supplemented with 10% FBS for 12 hours and nearly 100% of oocytes extruded first polar bodies. High concentration (≥2.5 mg/ml) of myc_6_-Sgo1 mRNA was injected into cytoplasm and the oocytes were cultured for 2 hours before they were parthenogenetically activated by 10 mM SrCl_2._

Small interfering RNAs (siRNAs) of Sgo1 siRNAs (Ambion) were microinjected into cytoplasm to deplete Sgo1. The following 25 µM siRNAs was used. Sgo1-1 siRNA, GCUAACUUCCCGACAAAGUtt; Sgo1-2 siRNA, GCAUUGACAAAUACGACCAGtt; Sgo1-3 siRNA, CCAAAUUAGCUUAUGUUCUtt; or Sgo1-m siRNA, the mixed solution of the above three. The same amount of negative control siRNA (Qiagen) was also injected as control. After microinjection of GV oocytes, the oocytes were cultured for 24 hours in M16 supplemented with 10% FBS and 2.5 µM milrinone to prevent meiosis resumption.

### Quantification of RNAi effects in oocytes by real-time quantitative PCR

Analysis of relative gene expression was measured by real-time quantitative PCR and the 2(-Delta Delta C(T)) Method [29]. Total RNA and the first strand cDNA generation were performed as described above. cDNA fragment of Sgo1 and H2afz (H2A histone family, member Z, reference gene) [30] was amplified by the following primers. Sgo1, forward, TGGAGGTATTGGTTCCTGTGATG, reverse, CTGCATTCGAGGTCACTCACTTC. H2afz, forward, ACAGCGCAGCCATCCTGGAGTA, reverse, TTCCCGATCAGCGATTTGTGGA. Real-time PCR was used SYBR Premix Ex Taq™ kit (Takara) in ABI prism 7000 Sequence Detection System. The steps include 95°C 10s, 40 cycles of 95°C 5s and 60°C 31s.

### Immunofluorescent microscopy, chromosome spread and image analysis

Immunofluorescence was performed as described previously [31] except that oocytes were first left for 20 minutes in 1% sodium citrate in order to detect chromosomes clearly. Anti-myc-FITC antibody (Invitrogen) was applied at a dilution of 1∶300. For chromosome spreads, oocytes were left for 20 minutes in 1% sodium citrate at room temperature and then fixed by fresh methanol: glacial acetic acid (3∶1). 10 µg/ml PI was used for chromosome staining. Cells were examined with a Confocal Laser-Scanning Microscope (Zeiss LSM 510 META, Germany). Instrument settings were kept constant for each replicate.

### Statistical analysis

Data (mean±SE) were from at least three replicates per experiment and analyzed by ANOVA using SPSS software (SPSS Inc, Chicago, IL) followed by the student-Newman-Keuls test. Differences at P<0.05 were considered to be statistically significant.
